# The prognostic value of mitogen-activated protein kinase kinase in liver hepatocellular carcinoma by bioinformatics

**DOI:** 10.1097/MD.0000000000042933

**Published:** 2025-06-27

**Authors:** Shen Dong, Shen Jing, Jiao Qinshun, Wang Huaning, Zhu Rong

**Affiliations:** a Splenic-Gastric Disease Department, The First Affiliated Hospital of Yunnan University of Traditional Chinese Medicine (Yunnan Provincial Hospital of Traditional Chinese Medicine), Wuhua District, Kunming City, Yunnan Province, China; b Yunnan University of Chinese Medicine, Chenggong District, Kunming City, Yunnan Province, China.

**Keywords:** diagnostic markers, liver hepatocellular carcinoma, MAP2Ks family, prognostic markers

## Abstract

Liver hepatocellular carcinoma (LIHC) is a common cancer worldwide. Mitogen-activated protein kinase kinase (MAP2Ks) are related to the occurrence and development of a variety of tumors. However, the expression pattern, role, and prognostic value of the 7 MAP2K family members in LIHC have not yet been elucidated. We used the Oncomine, UALCAN, Human Protein Atlas, GeneMANIA, Gene Ontology, Kyoto Encyclopedia of Genes and Genomes, TIMER, and Kaplan–Meier Plotter databases. On August 7, 2021, we searched these databases for the terms MAP2K1, MAP2K2, MAP2K3, MAP2K4, MAP2K5, MAP2K6, MAP2K7, and “liver cancer.” The exposure group comprised LIHC patients, and the control group comprised normal patients (those with noncancerous liver tissue). All patients shown in the retrieval language search were included. We compared the mRNA expression of these proteins in LIHC and control patients to examine the potential role of MAP2K1 to 7 in LIHC. Relative to the normal liver tissue, mRNA expression of MAP2K1/3 was significantly downregulated (*P* < .001), MAP2K4 was downregulated (*P* < .05), and that of MAP2K2/5/6/7 significantly upregulated (*P* < .001), in LIHC. MAP2K mRNA expression varied with gender (*P* < .0001), cancer stage (*P* < .05), tumor grade (*P* < .05), and with node metastasis status (*P* < .05), except for MAP2K4. Based on Kyoto Encyclopedia of Genes and Genomes enrichment analysis, these genes were associated with the following pathways: MAPK signaling pathway, GnRH signaling pathway, Fc epsilon RI signaling pathway (*P* < .05). The MAP2Ks were significantly associated with purity (*P* < .05), except for MAP2K1/2, with B cell (*P* < .05), except for MAP2K3, and that all significantly associated withCD8+ T cell, CD4+ T cell, macrophage, neutrophil, and dendritic cell infiltration (*P* < .05). High mRNA expression of MAP2K1/3/4/5 (*P* < .05) and low expression of MAP2K6 (*P* < .05) indicated overall survival, the high expression of MAP2K3/4/5 were related to relapse free survival and progression free survival; the high expression of MAP2K3/5/7 were related to disease free survival. We identified MAP2K1 to 7 as potential diagnostic markers, and MAP2K2 to 7 as prognostic markers, of LIHC. Our future work will promote the use of MAP2Ks in the diagnosis and treatment of LIHC.

## 1. Introduction

Liver hepatocellular carcinoma (LIHC) is a common malignant tumor with extremely high morbidity and mortality. It is the 4th leading cause of cancer-related deaths in the world and has a serious impact on human life and health.^[1]^ Liver resection and liver transplantation are currently the most recommended treatments. Due to the insidious onset of LIHC, most patients are diagnosed at an intermediate to late stage and miss the best time for surgery.^[2,3]^ In addition, antiviral drugs and antitumor drugs are also common treatment means, but the efficacy of traditional anticancer drugs is affected by the resistance of anticancer drugs, limiting their full potential. For liver cancer caused by nonviral hepatitis, antiviral therapy seems to be useless. The difficulty of LIHC treatment and the limited treatment methods have become a consensus. Relevant studies have shown that the survival time of HCC patients within 5 years is <19%, the recurrence rate after treatment with conventional therapy can be as high as 80%^[4,5]^ The reasons for the high mortality of HCC patients include the difficulty of early diagnosis, the high degree of malignancy, the strong heterogeneity and the rapid progression of HCC. Therefore, early detection and treatment have become the key point to prolong the survival of LIHC patients. The diagnosis of HCC usually includes serum marker examination, imaging examination, and needle biopsy. However, imaging examination has the characteristics of high cost, complex operation and inapplicability to some special population, so it is difficult to become a routine screening method. Liver biopsy is an invasive examination method, and the patient’s acceptance of this method is doubtful, and due to the limited location of the biopsy, there may be missed diagnosis. Therefore, serum marker examination has become one of the most suitable screening methods. At present, the most commonly used marker of LIHC detection is AFP, although it has a certain predictive effect on the occurrence of disease, but for small liver cancer (<3 cm) were low, and 52% of small liver cancer patients were negative for AFP.^[6]^ The emergence of molecular targeted therapy has the possibility to expand and supplement LIHC treatment methods. As an emerging treatment method, it treats tumors through the characteristics of targeted targeting, which has attracted the attention and research of medical workers.^[7,8]^ Therefore, looking for effective diagnostic and prognostic markers has important implications for LICH.

Mitogen-activated protein kinase kinase (MAP2K) is a subtype family of mitogen-activated protein kinases. It is an indispensable part of the cascade signal reaction of the 3 kinases in the MAPK pathway. It is generally believed that phosphorylation of MAP3K activates MAP2K, and rephosphorylation of MAP2K activates MAPK, thereby participating in biological processes.^[9]^ Studies have shown that members of the MAP2Ks family are related to the occurrence and development of a variety of tumors, such as lung cell carcinoma, colorectal cancer, clear cell renal cell carcinoma, etc.^[10–12]^ In addition, recent studies have shown that some members of the MAP2Ks family are related to the proliferation and migration of LIHC.^[13–17]^ MAP2K1 (also known as MEK1) is a key signaling molecule involved in the regulation of a variety of cellular functions by downstream activation of mitogen-activated protein kinase pathways. Experimental studies have shown that overexpression of activated MAP2K1 can enhance the proliferation of cancer cells and confer drug resistance on cells. When MAP2K1 is knocked down, the migration ability of HepG2 cells is also inhibited.^[18]^ MAP2K2 (MEK2) plays a key role in mitogen growth factor signaling. Through their phosphorylation, MAPK1/ERK2 and MAPK3/ERK1 are activated. Drugs that inhibit MEK1/2 or ERK1/2 kinases can, through synergistic inhibition of ERK kinase activity, reverse liver cancer cell lines that do not respond to sorafenib in vitro and in vivo, increasing resistance to sorafenib.^[19]^ MAP2K3 (MKK3) and MAP2K6 (MKK6) can directly catalyze downstream p38MAPK, and then up-regulate the expression of MMP-9 through transcription factors such as AP1 and Sp1, leading to the migration and migration of tumor cells such as liver cancer.^[20]^ MAP2K4 (MKK4) and MAP2K7 (MKK7) are associated with stress-activating activities and biological events mediated by the c-Jun N-terminal kinase (JNK) signaling pathway. In the human body, MKK7 controls essential physiological processes, including but not limited to the proliferation and differentiation of multiple tissues and organs. Studies have shown that overexpressed RACK1 can enhance the activity of MKK7 and JNK by directly binding to MKK7, thus promoting the growth of liver cancer cells.^[21,22]^ However, there are few studies on the relationship between MAP2Ks and LIHC in terms of expression patterns and prognosis.

In the present study, we investigated the relationship between MAP2Ks and LIHC from the perspective of bioinformatics. We searched for the transcription and protein expression of MAP2Ks in LIHC, predicted the gene ontology functions and biological pathways of MAP2Ks and its 20 related genes, and analyzed the prognostic value of MAP2Ks in LIHC between different subgroups. Our findings provide assistance in the diagnosis and treatment of LIHC.

## 2. Methods

### 2.1. Ethical approval

These analyses were based on online open-access databases; hence this article does not contain any research conducted by any author on human participants or animals, nor can it be followed up and updated.

### 2.2. Oncomine database

Oncomine (http://www.oncomine.org) is a cancer microarray database for genome-wide expression analysis. Seven hundred fifteen datasets (86,733 samples) were selected.^[23]^ The mRNA differential expressions of 7 MAP2Ks family members in LIHC tissues were analyzed online. The fold change was as follows: *P* < .05, fold change = 1.5, gene grade = 10%.

### 2.3. UALCAN database

UALCAN (http://ualcan.path.uab.edu) is a network database of TCGA database RNA-seq and 31 cancer types.^[24]^ The mRNA expression patterns of MAP2Ks family members in LIHC tissues (N = 371) and normal tissues (N = 50) were compared in the data set, and subgroup analysis were performed. The subgroups were set as gender, grade, stage, and lymph node metastasis status.

### 2.4. Human Protein Atlas

The Human Protein Atlas (HPA, http://www.proteinatlas.org) collects representative immunohistochemistry-based protein expression data for nearly 20 highly common cancers.^[25]^ Immunohistochemically analysis images of MAP2Ks family member protein expression in clinical specimens of LIHC patients and normal tissues were obtained from the HPA database. We selected HPA records with *P* < .05.

### 2.5. GeneMANIA database

GeneMANIA (http://www.genemania.org) is a website that uses available genomics and proteomics data to generate hypotheses about gene function.^[26]^ We assumed the top 20 related genes of MAP2Ks and visualized the functional association network of MAP2Ks members with them. Advanced statistical options: maximum synthetic attribute = 10, maximum synthetic gene = 20. GeneMANIA considers *P* < .05 statistically significant.

### 2.6. GO and KEGG enrichment analysis

The Gene Ontology database (GO, http://geneontology.org) comprehensively describes the properties of genes and gene products in organisms by describing the molecular functions of genes, the role of cell components, and the biological processes involved.^[27]^ The Kyoto Encyclopedia of Genes and Genomes (KEGG, http://www.kegg.jp) is a database that integrates information on genome, chemistry, and system functions.^[28]^ We used bioconductor for GO and KEGG enrichment analysis.

### 2.7. Timer database

TIMER (https://cistrome.shinyapps.io/timer) is a systematic database that uses microarrays to express values. TIMER algorithm estimates the abundance of the 6 immune infiltrations (B cells, CD4+ T cells, CD8+ T cells, neutrophils, macrophages, and dendritic cells). It can be used to evaluate the immune infiltration of genes in cancer.^[29]^ The immune infiltration of MAP2Ks in LIHC were evaluated through TIMER. Statistical significance was set at *P* < .05.

### 2.8. Kaplan–Meier plotter

Kaplan–Meier plotter (http://kmplot.com/analysis) is an online database that analyzes the prognosis of different types of cancer.^[30]^ We assessed the overall survival (OS), relapse free survival (RFS), progression free survival (PFS), and disease free survival (DSS) of 364 LIHC patients to clarify the prognostic value of mRNA expression of each MAP2Ks member and perform subgroup analysis. The subgroup settings were: Gender, Grade, Stage, American Joint Committee on Cancer (AJCC)_T, and Vascular invasion. *P* < .05 was considered statistically significant.

### 2.9. Statistical analysis

SPSS 25.0 (IBM, Armonk) was used for statistical analysis. Results were considered significant at *P* < .05. For Cox proportional hazard regression analysis, the 95% confidence interval and hazard ratio were used for risk assessment.

### 2.10. Data management and collection

We obtained records from the Oncomine, UALCAN, HPA, TIMER, and Kaplan–Meier plotter databases on August 7, 2021. Records were obtained by searching these databases using the terms MAP2K1, MAP2K2, MAP2K3, MAP2K4, MAP2K5, MAP2K6, MAP2K7, and “liver cancer.” The exposure group comprised patients with LIHC, and the control group comprised normal (noncancerous) tissue samples. All patients included in the search language search were included. The search was not restricted based on race, country, gender, or language. Two researchers (SD and SJ) independently reviewed the eligibility of the data, and WS resolved any discrepancies. Disagreements over eligibility were resolved via discussion. The research selection process conformed to the STROBE guidelines. To ensure the validity and reliability of the results, SD independently conducted statistical analysis. WHN reviewed the data to detect potential bias that could arise during subgroup analysis.

## 3. Results

### 3.1. mRNA expression of MAP2Ks in LIHC patients

**Figure 1.**
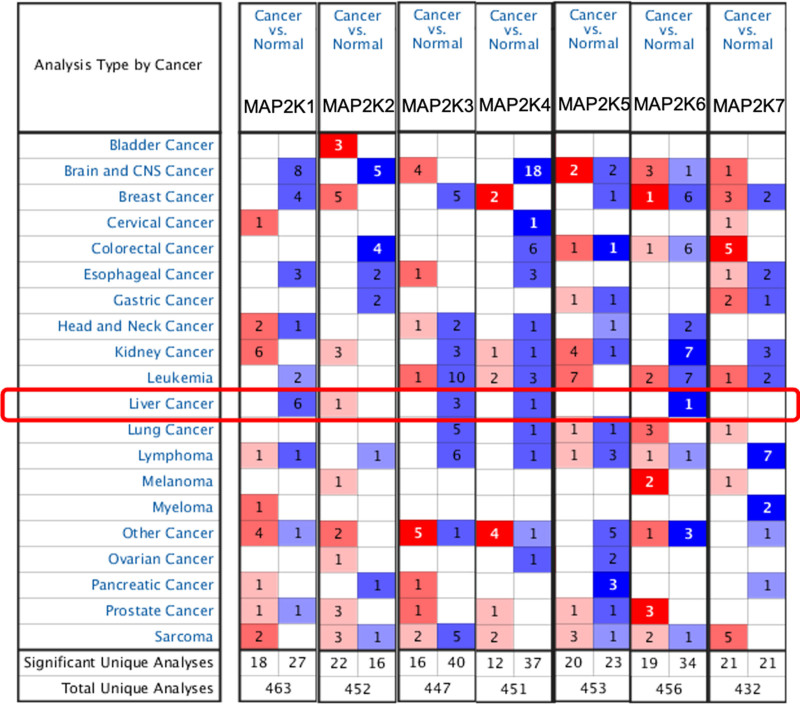
mRNA expression levels of MAP2Ks in 20 types of cancer. Cutoff *P* value and fold change: *P* value < .05, fold change = 1.5, gene grade = 10%. Red means high expression, blue means low expression. MAP2K = mitogen-activated protein kinase kinase.

Using the Oncomine database, we compared MAP2K transcription in 20 cancers and normal tissues: mRNA expression of MAP2K1/3/4/6 was significantly lower, and MAP2K2 was significantly higher, in LIHC tissue (*P* < .05) (Fig. 1 and Table 1).

**Table 1:** mRNA expression level of MAP2Ks between LIHC and normal liver tissue (Oncomine).

MAP2Ks	Types of LIHC	Fold change	*P*-value	*t* test	Ref
MAP2K1	Hepatocellular carcinoma	‐3.189	1.85E‐5	‐6.800	Wurmbach Liver^[25]^
	Cirrhosis	‐2.059	7.71E‐4	‐4.026	Wurmbach Liver^[25]^
	Hepatocellular carcinoma	‐1.583	3.11E‐11	‐7.074	Chen Liver^[27]^
	Hepatocellular carcinoma	‐1.516	2.23E‐29	‐12.061	Roessler Liver 2^[28]^
	Hepatocellular carcinoma	‐1.589	1.66E‐5	‐4.683	Roessler Liver^[28]^
	Hepatocellular carcinoma	‐2.053	9.39E‐7	‐6.104	Mas Liver^[26]^
MAP2K2	Hepatocellular carcinoma	1.769	1.41E‐5	4.772	Roessler Liver^[28]^
MAP2K3	Hepatocellular carcinoma	‐1.601	8.96E‐8	‐6.417	Roessler Liver^[28]^
	Hepatocellular carcinoma	‐1.676	3.64E‐41	‐14.872	Roessler Liver 2^[28]^
	Hepatocellular carcinoma	‐1.856	1.03E‐12	‐7.808	Chen Liver^[27]^
MAP2K4	Hepatocellular carcinoma	‐1.782	2.81E‐14	‐8.285	Chen Liver^[27]^
MAP2K6	Hepatocellular carcinoma	‐2.162	1.88E‐5	5.430	Wurmbach Liver^[25]^

**Figure 2.**
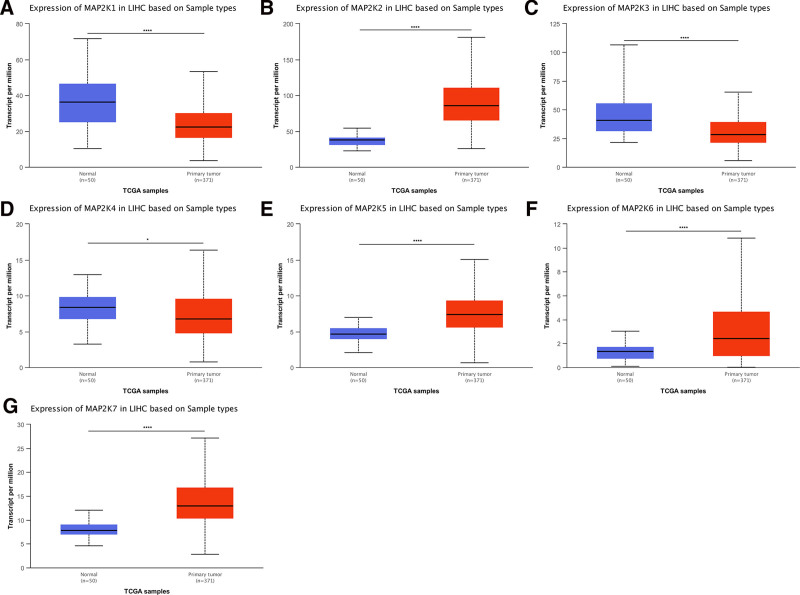
mRNA expression levels of MAP2Ks between LIHC and normal liver tissues (UALCAN database). LIHC = liver hepatocellular carcinoma, MAP2K = mitogen-activated protein kinase kinase.

We then verified these results using the UALCAN database. Relative to normal liver tissue, mRNA expression of MAP2K1/3 was significantly downregulated (*P* < .001), MAP2K4 was downregulated (*P* < .05), and that of MAP2K2/5/6/7 significantly upregulated (*P* < .001), in LIHC (Fig. 2A–G).

**Figure 3.**
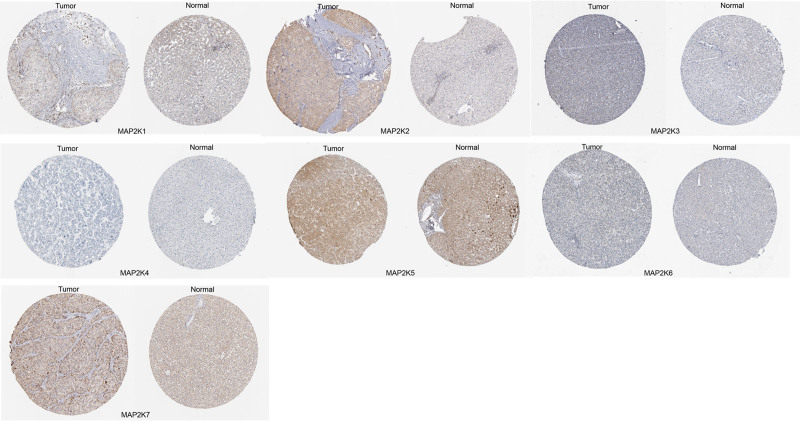
Protein expression of MAP2Ks between LIHC and normal liver tissues (HPA database). HPA = Human Protein Atlas, LIHC = liver hepatocellular carcinoma, MAP2K = mitogen-activated protein kinase kinase.

The protein expression of MAP2Ks in LIHC were examined in HPA database (Fig. 3). The results showed that MAP2K1/2/3/4 were not detected in the liver, low expression of MAP2K5/6/7 was observed in the liver, while low expression of MAP2K1/3 and high expression of MAP2K2 was detected in LIHC; MAP2K4 was not detected in LIHC, the expression of MAP2K5/7 was found in LIHC.

### 3.2. Subgroup analysis of MAP2Ks mRNA expression in LIHC

In order to explore the differences in the mRNA expression of MAP2Ks family members between LIHC and normal liver tissues, subgroup analysis was used to study them in groups from gender, individual tumor stage, tumor grade, and lymphatic metastasis status.

#### 3.2.1. MAP2Ks mRNA expression in LIHC by gender

**Figure 4.**
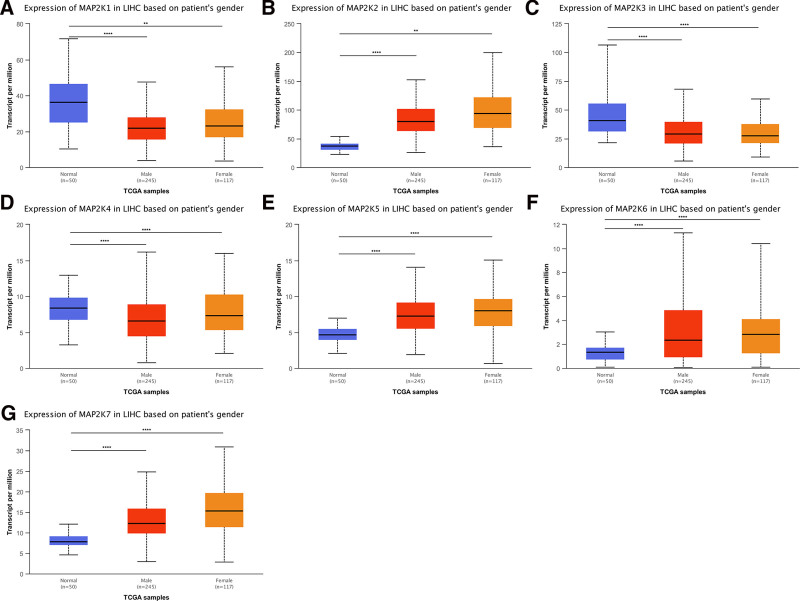
mRNA expression levels of MAP2Ks in LIHC patients with different gender subtypes (UALCAN database). LIHC = liver hepatocellular carcinoma, MAP2K = mitogen-activated protein kinase kinase.

We compared 50 patients with normal (noncancerous) liver tissue, 245 male LIHC patients, and 117 female LIHC patients: In male patients, low expression of MAP2K1/3/4 were observed significantly (*P* < .0001) (Fig. 4A, C and D); high expression of MAP2K2/5/6/7 were detected significantly (*P* < .0001) (Fig. 4B, E–G). In female patients, MAP2K1 was low expression (*P* < .01); MAP2K3/4 were significantly low expression (*P* < .0001) (Fig. 4A, C, D); MAP2K2 was high expression (*P* < .01); MAP2K5/6/7 were significantly high expression (*P* < .0001) (Fig. 4B, E–G).

#### 3.2.2. MAP2Ks mRNA expression in LIHC by cancer stage

**Figure 5.**
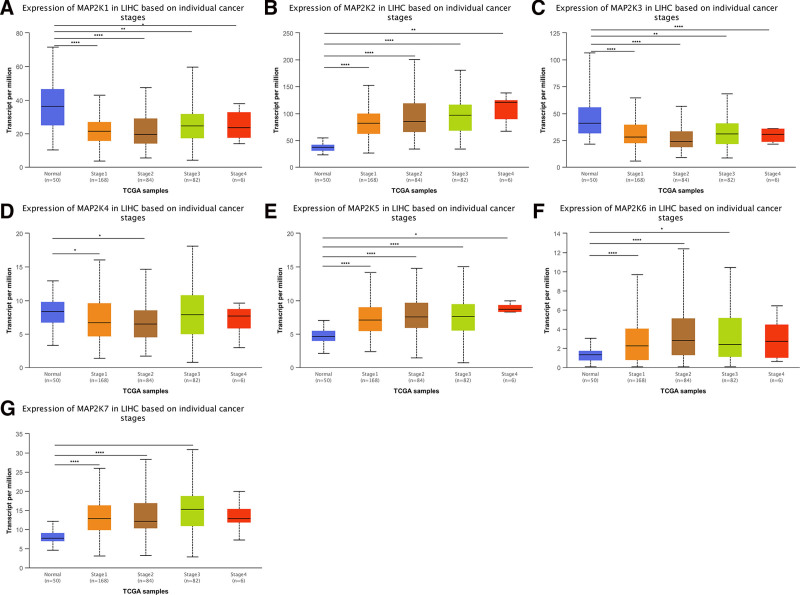
mRNA expression levels of MAP2Ks in LIHC patients with different tumor stages (UALCAN database). LIHC = liver hepatocellular carcinoma, MAP2K = mitogen-activated protein kinase kinase.

We compared 50 patients with normal liver tissue with 168 stage 1, 84 stage 2, 82 stage 3, and 6 stage 4 LIHC patients: MAP2K1/3 were low expressed in all tumor stages (*P* < .05, Fig. 5A and C). MAP2K2/5 were high expression in all tumor stages (*P* < .05, Fig. 5B and E). MAP2K4 was low expressed in stage1 and stage2 (*P* < .05, Fig. 5D). MAP2K6/7 were significantly high expression in stage1/2 (*P* < .0001) and were high expression in stage3 (*P* < .05) (Fig. 5F and G).

#### 3.2.3. MAP2Ks mRNA expression in LIHC by tumor grade

**Figure 6.**
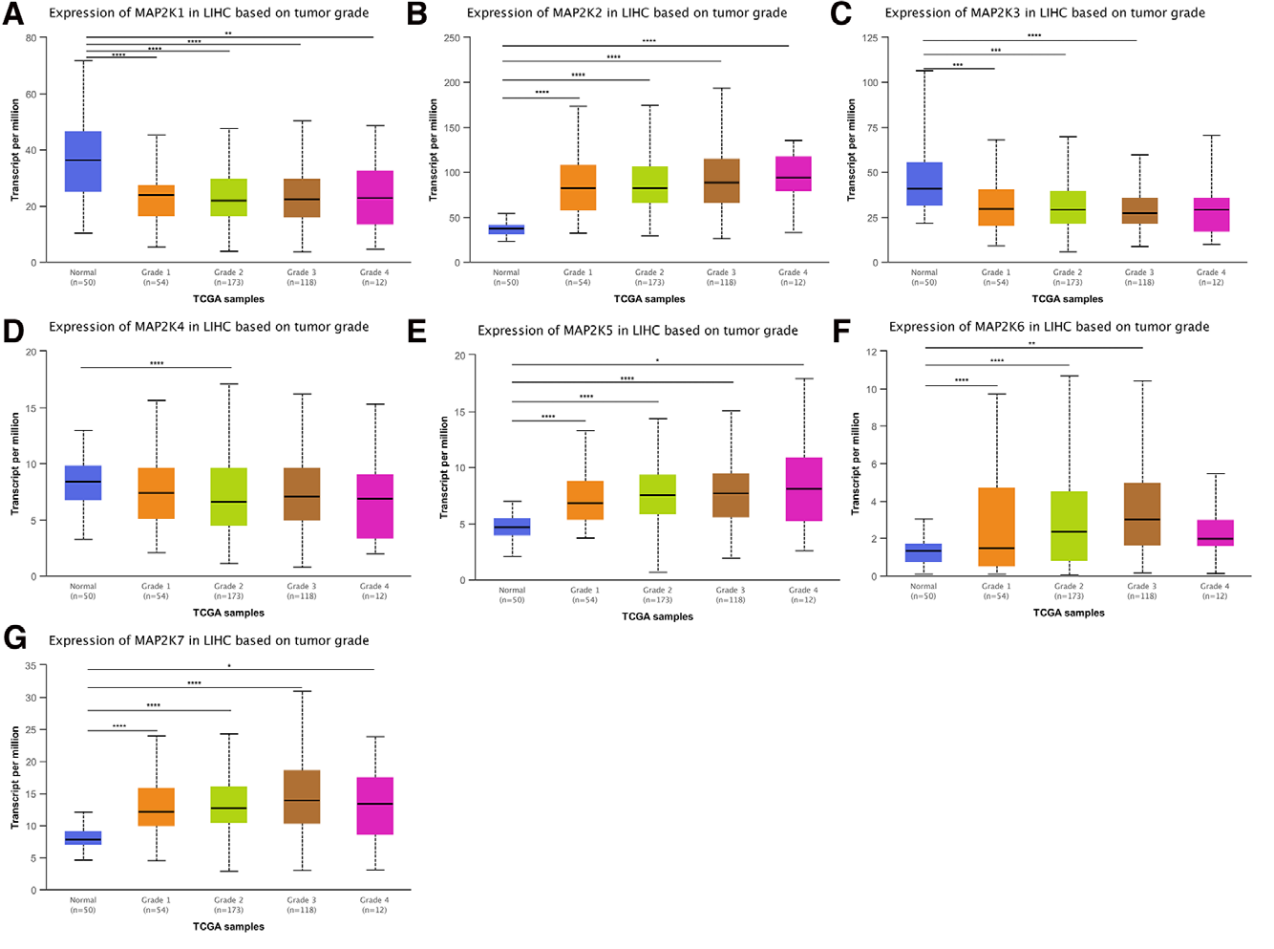
mRNA expression levels of MAP2Ks in LIHC patients with different tumor grades (UALCAN database). LIHC = liver hepatocellular carcinoma, MAP2K = mitogen-activated protein kinase kinase.

We compared mRNA expression in 50 patients with normal (noncancerous) liver tissue, 54 grade 1 LIHC patients, 173 grade 2 patients, 118 grade 3 patients, and 12 grade 4 patients: MAP2K1 was low expressed in all grades (*P* < .01, Fig. 6A); MAP2K2/5/7 were high expressed in all grades (*P* < .05, Fig. 6B, E, and G); MAP2K3 was low expressed in grade1 to 3 (*P* < .001, Fig. 6C); MAP2K4 was low-expressed in grade2 (*P* < .0001, Fig. 6D); MAP2K6 was high expressed in grade1 to 3 (*P* < .001, Fig. 6F).

#### 3.2.4. MAP2Ks mRNA expression in LIHC by node metastasis status

**Figure 7.**
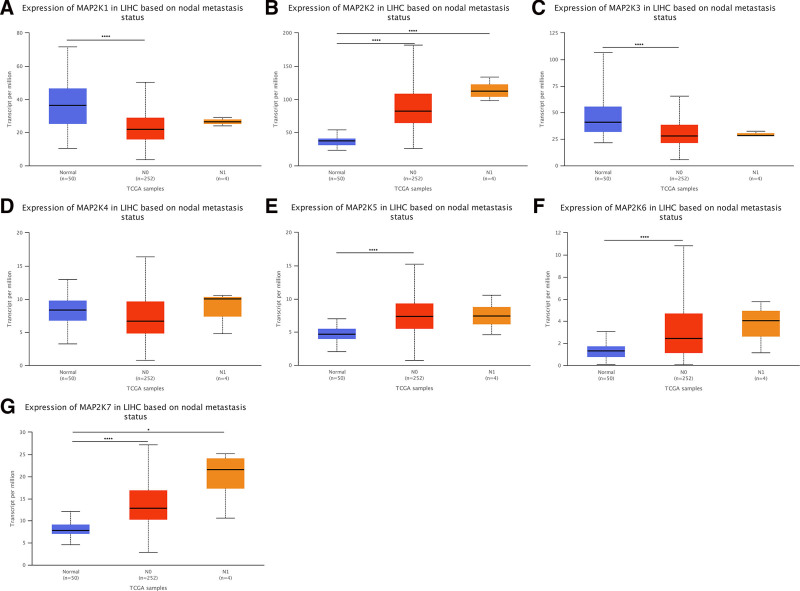
mRNA expression levels of MAP2Ks family members in different lymphatic metastasis states of LIHC (UALCAN database). LIHC = liver hepatocellular carcinoma, MAP2K = mitogen-activated protein kinase kinase.

We compared 50 patients with normal liver tissue, with 252 node metastasis status N0 and 4 N1 status LIHC patients. In patients with lymphatic metastasis N0, MAP2K1/3 showed low expression (*P* < .0001, Fig. 7A and C); MAP2K2/5 to 7 showed high expression (*P* < .0001, Fig. 7B and E–G). In patients with lymphatic metastasis N1, MAP2K2/7 were high expressed (*P* < .05, Fig. 7B and G). MAP2K4 was not statistically significant in patients with lymphatic metastasis N0 and N1 (*P* > .05, Fig. 7D).

### 3.3. Functional enrichment of MAP2Ks in LIHC

**Figure 8.**
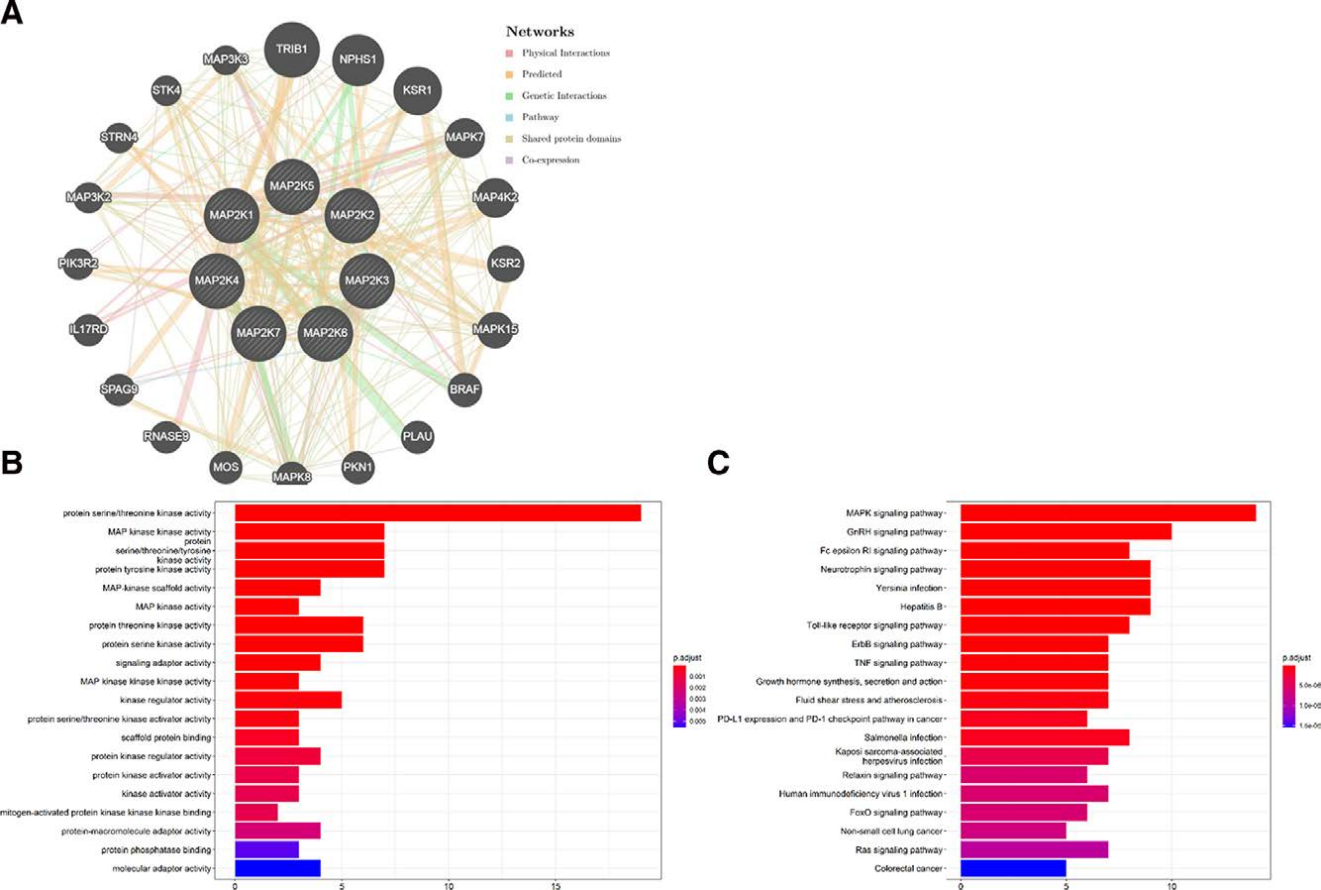
GO enrichment and KEGG pathway analysis of MAP2Ks protein interaction. (A) GeneMANIA constructed a network of MAP2Ks and 20 related genes. (B) GO enrichment analysis ranks the top 20 functions; (C) KEGG pathway analysis ranked the top 20 pathways. GO = Gene Ontology database, KEGG = Kyoto Encyclopedia of Genes and Genomes, MAP2K = mitogen-activated protein kinase kinase.

In order to explore the closely related genes of MAP2Ks family members and their functional modes of action, we constructed a network of related genes through GeneMANIA (Fig. 8A). The results showed that the top 20 genes closely related to MAP2Ks are: TRIB1, NPHS1, KSR1, MAPK7, MAP4K2, KSR2, MAPK15, BRAF, PLAU, PKN1, MAPK8, MOS, RNASE9, SPAG9, IL17RD, PIK3R2, MAP3K2, STRN4, STK4, and MAP3K3.

The GO function and pathway of MAP2Ks and 20 related genes were analyzed by bioconductor. As shown in Fig. 8B, the top 20 functions and approaches are GO:0004674 (protein serine/threonine kinase activity), GO:0004708 (MAP kinase kinase activity), GO:0004712 (protein serine/threonine/tyrosine kinase activity), GO:0004713 (protein tyrosine kinase activity), GO:0005078 (MAP-kinase scaffold activity), GO:0004707 (MAP kinase activity), GO:0106311 (protein threonine kinase activity), GO:0106310 (protein serine kinase activity), GO:0035591 (signaling adaptor activity), GO:0004709 (MAP kinase kinase kinase activity), GO:0019207 (kinase regulator activity), GO:0043539 (protein serine/threonine kinase activator activity), GO:0097110 (scaffold protein binding), GO:0019887 (protein kinase regulator activity), GO:0030295 (protein kinase activator activity), GO:0019209 (kinase activator activity), GO:0031435 (mitogen-activated protein kinase kinase kinase binding), GO:0030674 (protein-macromolecule adaptor activity), GO:0019903 (protein phosphatase binding), and GO:0060090 (molecular adaptor activity).

KEGG enrichment analysis results show (Fig. 8C), the top 20 KEGG pathways are hsa04010 (MAPK signaling pathway), hsa04912 (GnRH signaling pathway), hsa04664 (Fc epsilon RI signaling pathway), hsa04722 (Neurotrophin signaling pathway), hsa05135 (Yersinia infection), hsa05161 (Hepatitis B), hsa04620 (Toll-like receptor signaling pathway), hsa04012 (ErbB signaling pathway), hsa04668 (TNF signaling pathway), hsa04935 (growth hormone synthesis, secretion, and action), hsa05418 (fluid shear stress and atherosclerosis), hsa05235 (PD-L1 expression and PD-1 checkpoint pathway in cancer), hsa05132 (Salmonella infection), hsa05167 (Kaposi sarcoma-associated herpesvirus infection), hsa04926 (relaxin signaling pathway), hsa05170 (human immunodeficiency virus 1 infection), hsa04068 (FoxO signaling pathway), hsa05223 (non-small cell lung cancer), Hsa0401 (Ras signaling pathway), and hsa05210 (colorectal cancer).

### 3.4. Correlation between MAP2Ks mRNA expression and LIHC immune infiltration

**Figure 9.**
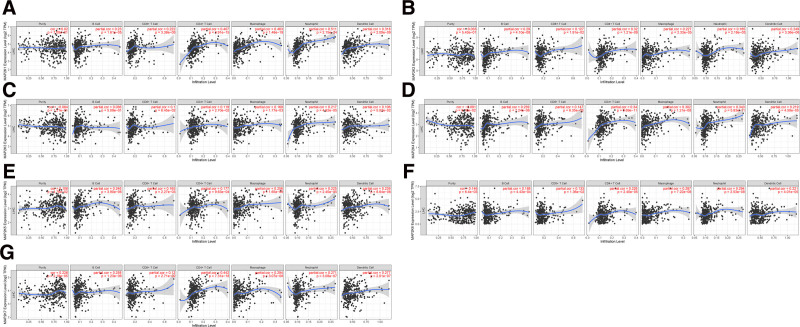
Correlation analysis of the level of immune infiltration of MAP2Ks in LIHC patients. The mRNA expression of MAP2Ks was significantly correlated with the level of immune infiltration in LIHC (A–G). LIHC = liver hepatocellular carcinoma, MAP2K = mitogen-activated protein kinase kinase.

The relationship between the mRNA expression of MAP2Ks and the level of immune infiltration in LIHC were analyzed using the data in the TIMER database. The results showed MAP2K3 to 7 were significantly correlated with tumor purity (Fig. 9C–G). Except for MAP2K3, all of MAP2Ks family is statistically significant with that of B cells (Fig. 9A, B and D–G). all MAP2Ks family members was significantly correlated with the infiltration of CD8+ T, CD4+ T cells, macrophages, neutrophils and dendritic cells (Fig. 9A–G).

### 3.5. The prognostic value of MAP2Ks in LIHC patients

**Figure 10.**
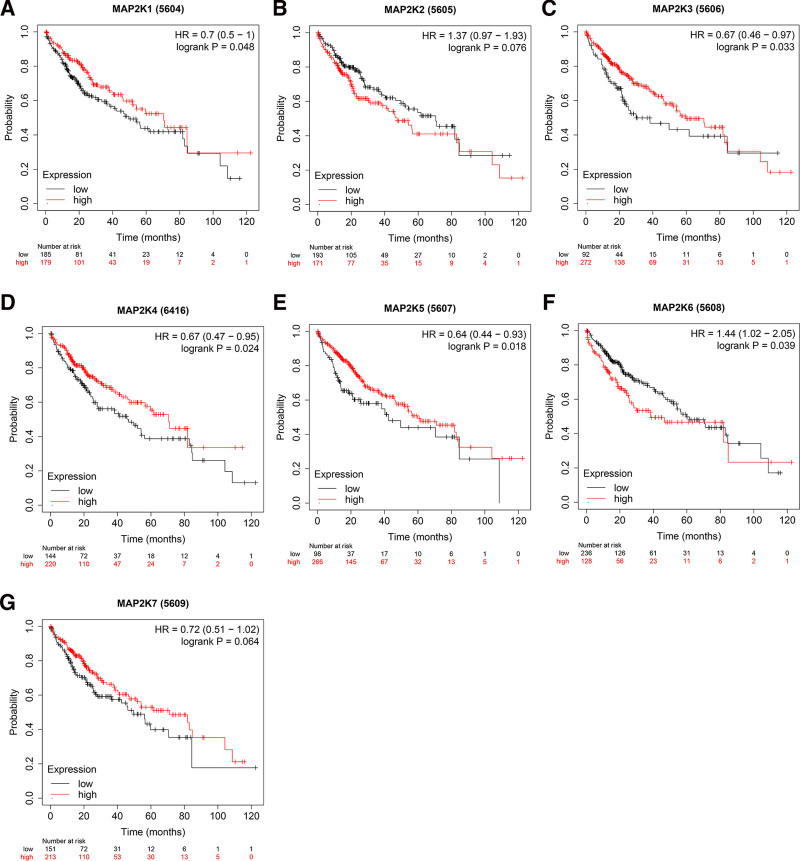
The prognostic value (OS) of MAP2Ks in LIHC patients. Comparation of the Overall Survival of high and low expression of MAP2Ks family members of LIHC patients (Kaplan–Meier plotter). LIHC = liver hepatocellular carcinoma, MAP2K = mitogen-activated protein kinase kinase.

**Figure 11.**
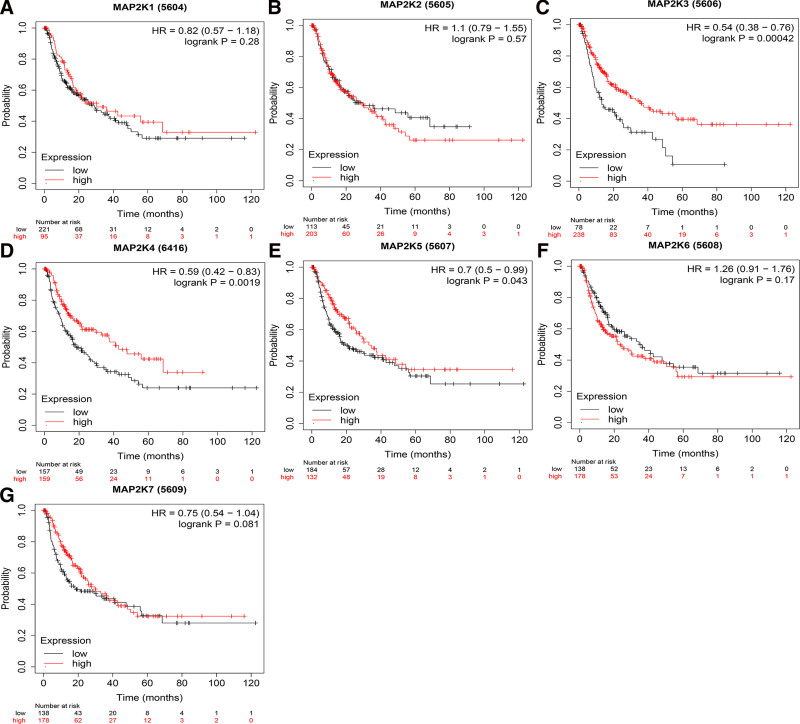
The prognostic value (RFS) of MAP2Ks in LIHC patients. Comparison of the relapse free survival between the high expression and low expression of MAP2Ks family members of LIHC patients (Kaplan–Meier plotter). LIHC = liver hepatocellular carcinoma, MAP2K = mitogen-activated protein kinase kinase.

**Figure 12.**
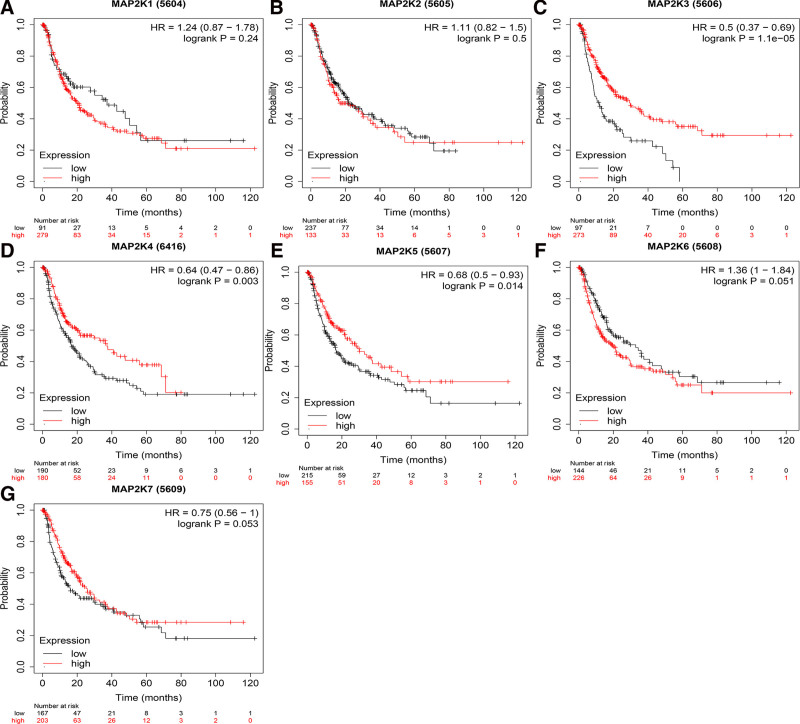
The prognostic value (PFS) of MAP2Ks in LIHC patients. Comparison of progression free survival between high expression and low expression of MAP2Ks family members in LIHC patients (Kaplan–Meier plotter). LIHC = liver hepatocellular carcinoma, MAP2K = mitogen-activated protein kinase kinase, PFS = progression free survival.

**Figure 13.**
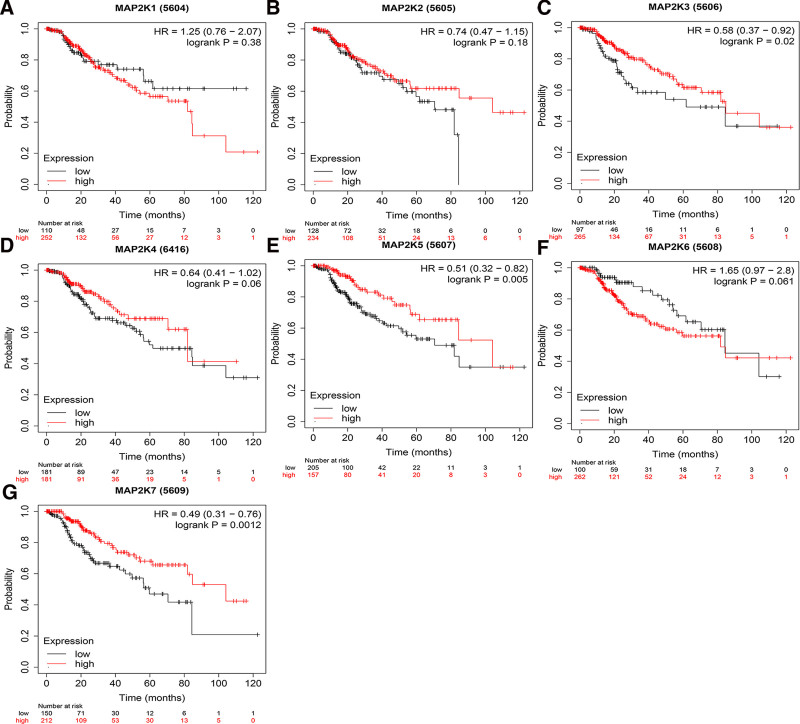
The prognostic value (DSS) of MAP2Ks in LIHC patients. Comparison of the high and low expression (PFS) and disease free survival of MAP2Ks family members in LIHC patients (Kaplan–Meier plotter). LIHC = liver hepatocellular carcinoma, PFS = progression free survival.

The correlation between MAP2Ks and the prognosis of LIHC patients were examined. The results showed that the high expression of MAP2K1/3/4/5 and the low expression of MAP2K6 were related to the OS of LIHC patients (*P* < .05, Fig. 10A and C–F). MAP2K2/7 were not associated with OS of LIHC patients (*P* > .05, Fig. 10B and G). The high expression of MAP2K3/4/5 were correlated with RFS and PFS of LIHC patients (*P* < .05, Figs. 11C–E and 12C–E). MAP2K1/2/6/7 was not associated with RFS or PFS in LIHC patients (*P* > .05, Figs. 11A, B, F, G and 12A, B, F, G). The high expression of MAP2K3/5/7 were associated with DSS in LIHC patients (*P* < .05, Fig. 13C, E, and G). MAP2K1/2/4/6 was not associated with DSS in LIHC patients (*P* > .05, Fig. 13A, B, D, and F).

The prognostic value of OS, RFS, PFS, and DSS of MAP2Ks in patients with different subtypes of LIHC was further analyzed by subgroup analysis. As shown in Table 2, elevation of MAP2K1 was associated with OS in male patients; MAP2K2 was not associated with the prognosis of male and female patients; elevated MAP2K3/4 was associated with OS, RFS, PFS, and DSS in male patients, elevation of MAP2K3 was associated with PFS in female patients; the increase of MAP2K5 was associated with OS, PFS, and DSS in male patients; the decrease of MAP2K6 was associated with OS, RFS, and DSS in female patients; the increase of MAP2K7 was associated with OS and RFS in female patients.

**Table 2:** Analysis of the prognostic value of MAP2Ks in LIHC patients of different genders.

MAP2Ks	Gender	OS			RFS			PFS			DSS		
		CASES	HR (95% CI)	*P*	CASES	HR (95% CI)	*P*	CASES	HR (95% CI)	*P*	CASES	HR (95% CI)	*P*
MAP2K1	MALE	246	**0.6 (0.38–0.95**)	**.0263**	208	0.8 (0.5–1.27)	.34	246	0.76 (0.5–1.15)	.19	241	1.37 (0.76–2.46)	.29
	FEMALE	118	1.44 (0.79–2.62)	.2348	105	0.73 (0.4–1.31)	.28	120	0.76 (0.46–1.27)	.3	116	1.47 (0.68–3.15)	.32
MAP2K2	MALE	246	1.36 (0.87–2.13)	.1716	208	0.84 (0.52–1.35)	.47	246	1.12 (0.78–1.63)	.54	241	0.66 (0.37–1.18)	.16
	FEMALE	118	1.5 (0.86–2.61)	.1519	105	1.39 (0.74–2.63)	.3011	120	1.36 (0.79–2.34)	.26	116	1.64 (0.79–3.39)	.18
MAP2K3	MALE	246	**0.54 (0.34–0.86**)	**.0085**	208	**0.5 (0.33–0.77**)	**.0011**	246	**0.49 (0.34–0.72**)	**.00015**	241	**0.48 (0.27–0.84**)	**.0094**
	FEMALE	118	1.48 (0.84–2.59)	.1688	105	0.58 (0.31–1.07)	.078	120	**0.51 (0.3–0.86**)	**.011**	116	0.53 (0.26–1.09)	.081
MAP2K4	MALE	246	**0.49 (0.31–0.77**)	**.0017**	208	**0.46 (0.31–0.69**)	**.0001**	246	**0.55 (0.38–0.79**)	**.00092**	241	**0.5 (0.28–0.89**)	**.016**
	FEMALE	118	0.73 (0.39–1.36)	.3202	105	0.59 (0.32–1.09)	.089	120	0.64 (0.38–1.08)	.093	116	1.41 (0.65–3.08)	.38
MAP2K5	MALE	246	**0.63 (0.4–0.98**)	**.038**	208	0.68 (0.44–1.06)	.087	246	**0.62 (0.42–0.93**)	**.019**	241	**0.47 (0.26–0.83**)	**.0077**
	FEMALE	118	0.66 (0.37–1.2)	.172	105	0.68 (0.37–1.27)	.23	120	0.74 (0.43–1.25)	.26	116	0.56 (0.26–1.21)	.14
MAP2K6	MALE	246	1.68 (0.97–2.97)	.0609	208	0.76 (0.49–1.16)	.1968	246	1.41 (0.94–2.11)	.096	241	1.84 (0.94–3.61)	.073
	FEMALE	118	**2.18 (1.2–3.95**)	**.0084**	105	**1.86 (1.01–3.4**)	**.042**	120	1.56 (0.93–2.61)	.089	116	**2.4 (1.12–5.17**)	**.021**
MAP2K7	MALE	246	0.67 (0.43–1.04)	.073	208	0.74 (0.48–1.14)	.1736	246	0.7 (0.47–1.04)	.079	241	**0.4 (0.23–0.72**)	**.0014**
	FEMALE	118	**0.55 (0.31–0.97**)	**.036**	105	**0.56 (0.31–1.01**)	**.0498**	120	0.61 (0.36–1.03)	.064	116	0.5 (0.23–1.08)	.073

Bold values indicate *P* < .05.

CI = confidence interval, HR = hazard ratio, OS = overall survival, PFS = progression free survival, RFS = relapse free survival.

As shown in Table 3, the increase of MAP2K1 was related to Grade1’s RFS, PFS, and Grade1 to 2 OS, and its decrease was related to Grade1’s DSS, Grade3’s RFS and PFS. The increase of MAP2K2 was related to the RFS and PFS of Grade3, and the decrease of MAP2K2 was related to the OS and DSS. The increase in MAP2K3 was related to the OS of Grade1, the PFS of Grade2, and the RFS and PFS of Grade3. The increase in MAP2K4 was related to the OS and PFS of Grade 1, the RFS and PFS of Grade 2, and the OS and RFS of Grade 3; the increase of MAP2K5 was related to the RFS and PFS of Grade 2, and the OS of Grade 3; the increase of MAP2K6 was related to the RFS and PFS of Grade 1, Its decrease was related to the OS and DSS of Grade 2 and the RFS and PFS of Grade 3; the increase of MAP2K7 was related to the PFS and DSS of Grade 2 and the DSS of Grade 3.

**Table 3:** Analysis of the prognostic value of MAP2Ks in LIHC patients of different grades.

MAP2Ks	Grade	OS			RFS			PFS			DSS		
		CASES	HR (95% CI)	*P*	CASES	HR (95% CI)	*P*	CASES	HR (95% CI)	*P*	CASES	HR (95% CI)	*P*
MAP2K1	1	55	2.1 (0.83–5.35)	.1114	45	**0.18 (0.06–0.53**)	**.00049**	55	**0.22 (0.09–0.53**)	**.00022**	55	**3.21 (0.97–10.62**)	**.044**
	2	174	**0.59 (0.35–0.98**)	**.0399**	147	1.24 (0.73–2.11)	.43	175	1.34 (0.87–2.07)	.18	169	0.76 (0.39–1.48)	.42
	3	118	**0.48 (0.23–0.99**)	**.0429**	106	**2.16 (1.18–3.95**)	**.011**	119	**1.76 (1.01–3.07**)	**.042**	116	0.73 (0.32–1.66)	.45
	4	12	NA	NA	11	NA	NA	12	NA	NA	12	NA	NA
MAP2K2	1	55	0.42 (0.12–1.5)	.1695	45	2 (0.75–5.33)	.16	55	1.98 (0.89–4.39)	.089	55	0.19 (0.02–1.55)	.088
	2	174	0.71 (0.41–1.24)	.2284	147	1.6 (0.95–2.67)	.072	175	1.48 (0.93–2.33)	.094	169	0.65 (0.32–1.31)	.22
	3	118	**2.12 (1.15–3.89**)	**.0133**	106	**0.43 (0.2–0.92**)	**.024**	119	**0.57 (0.35–0.94**)	**.026**	116	**2.13 (0.99–4.61**)	**.048**
	4	12	NA	NA	11	NA	NA	12	NA	NA	12	NA	NA
MAP2K3	1	55	**0.36 (0.14–0.95**)	**.0317**	45	0.63 (0.23–1.75)	.37	55	0.49 (0.21–1.11)	.083	55	0.4 (0.12–1.4)	.14
	2	174	0.72 (0.45–1.25)	.2404	147	0.66 (0.39–1.12)	.12	175	**0.52 (0.33–0.83**)	**.005**	169	0.6 (0.3–1.2)	.14
	3	118	0.56 (0.3–1.05)	.0664	106	**0.33 (0.19–0.59**)	**6.6e‐05**	119	**0.38 (0.23–0.65**)	**2e‐04**	116	0.51 (0.23–1.1)	.079
	4	12	NA	NA	11	NA	NA	12	NA	NA	12	NA	NA
MAP2K4	1	55	**0.36 (0.13–1.03**)	**.0459**	45	0.55 (0.21–1.44)	.22	55	**0.33 (0.15–0.72**)	**.0035**	55	0.29 (0.08–1.13)	.057
	2	174	1.52 (0.84–2.77)	.1641	147	**0.46 (0.25–0.84**)	**.0098**	175	**0.55 (0.33–0.91**)	**.019**	169	0.4 (0.15–1.04)	.051
	3	118	**0.42 (0.23–0.76**)	**.003**	106	**0.53 (0.3–0.91**)	**.019**	119	0.6 (0.36–1.01)	.054	116	0.43 (0.2–0.92)	.025
	4	12	NA	NA	11	NA	NA	12	NA	NA	12	NA	NA
MAP2K5	1	55	1.47 (0.58–3.74)	.4121	45	0.57 (0.2–1.63)	.29	55	0.69 (0.32–1.52)	.36	55	0.4 (0.11–1.39)	.13
	2	174	0.6 (0.35–1.02)	.059	147	**0.61 (0.37–1**)	**.047**	175	0.65 (0.42–1.01)	.054	169	**0.45 (0.22–0.94**)	**.028**
	3	118	**0.5 (0.27–0.93**)	**.0254**	106	0.74 (0.42–1.31)	.3	119	0.67 (0.39–1.14)	.14	116	0.47 (0.21–1.94)	.057
	4	12	NA	NA	11	NA	NA	12	NA	NA	12	NA	NA
MAP2K6	1	55	0.45 (0.17–1.17)	.092	45	**0.17 (0.04–0.75**)	**.008**	55	**0.39 (0.15–0.97**)	**.035**	55	0.59 (0.16–2.08)	.4
	2	174	**2 (1.18–3.41**)	**.0091**	147	1.24 (0.71–2.16)	.44	175	1.35 (0.84–2.18)	.21	169	**2.27 (1.15–4.46**)	**.015**
	3	118	1.52 (0.83–2.77)	.1721	106	**1.9 (1.04–3.47**)	**.033**	119	**1.82 (1.05–3.16**)	**.03**	116	0.68 (0.27–1.69)	.4
	4	12	NA	NA	11	NA	NA	12	NA	NA	12	NA	NA
MAP2K7	1	55	0.63 (0.2–1.94)	.41	45	2.02 (0.7–5.81)	.18	55	1.86 (0.86–4.05)	.11	55	0.22 (0.03–1.74)	.12
	2	174	0.59 (0.35–1.01)	.0521	147	0.67 (0.41–1.1)	.11	175	**0.6 (0.39–0.92**)	**.019**	169	**0.51 (0.26–1**)	**.046**
	3	118	0.73 (0.4–1.34)	.3092	106	0.67 (0.39–1.16)	.15	119	0.83 (0.5–1.37)	.46	116	**0.36 (0.15–0.84**)	**.014**
	4	12	NA	NA	11	NA	NA	12	NA	NA	12	NA	NA

Bold values indicate *P* < .05.

CI = confidence interval, HR = hazard ratio, OS = overall survival, PFS = progression free survival, RFS = relapse free survival.

As shown in Table 4, the increase of MAP2K1/5was related to the OS, RFS, PFS, and DSS of Stage3 + 4; the increase of MAP2K2 was related to the DSS of Stage2, the RFS and PFS of Stage3 + 4, and its decrease was related to the OS of Stage1. RFS of Stage2 and OS of Stage3 were related. The increase of MAP2K3 was related to the OS, RFS, PFS, and DSS of Stage1, and Stage3 + 4 DSS, and the decrease was related to the OS of Stage2 and the RFS of Stage3 + 4. The elevation of MAP2K4 was related to OS, RFS, and PFS of Stage1, OS, RFS, PFS, and DSS of Stage2, and RFS of Stage3 + 4. The increase of MAP2K5 was also related to the RFS and PFS of Stage1; the decrease of MAP2K6 was related to the OS and DSS of Stage2, and the RFS of Stage3 + 4; the increase of MAP2K7 was related to the RFS of Stage3 + 4, and its decrease were related to the OS of Stage1.

**Table 4:** Analysis of the prognostic value of MAP2Ks in LIHC patients at different stages.

MAP2Ks	Stage	OS			RFS			PFS			DSS		
		CASES	HR (95% CI)	*P*	CASES	HR (95% CI)	*P*	CASES	HR (95% CI)	*P*	CASES	HR (95% CI)	*P*
MAP2K1	1	170	0.6 (0.32–1.1)	.0942	153	0.67 (0.35–1.31)	.24	170	0.66 (0.36–1.2)	.17	167	0.59 (0.24–1.45)	.24
	2	83	0.46 (0.18–1.15)	.089	74	1.38 (0.66–2.9)	.39	84	1.74 (0.96–3.32)	.063	82	0.27 (0.06–1.24)	.072
	3 + 4	87	**0.57 (0.32–1.01**)	**.0495**	68	**0.53 (0.29–0.99**)	**.044**	88	**0.56 (0.33–0.96**)	**.034**	84	**0.46 (0.22–0.95**)	**.031**
MAP2K2	1	170	**2.08 (1.12–3.86**)	**.0176**	153	1.36 (0.76–2.42)	.29	170	0.72 (0.39–1.33)	.29	167	1.96 (0.81–4.77)	.13
	2	83	0.46 (0.21–1.02)	.0509	74	**2.04 (1.01–4.11**)	**.043**	84	0.78 (0.43–1.41)	.41	82	**0.21 (0.06–0.75**)	**.0081**
	3 + 4	87	**1.96 (1.09–3.53**)	**.023**	68	**0.45 (0.25–0.84**)	**.0093**	88	**0.57 (0.33–1**)	**.048**	84	1.83 (0.9–3.72)	.093
MAP2K3	1	170	**0.47 (0.26–0.87**)	**.0136**	153	**0.42 (0.24–0.73**)	**.0016**	170	**0.43 (0.26–0.72**)	**.0008**	167	**0.31 (0.12–0.77**)	**.0082**
	2	83	**2.34 (1.07–5.16**)	**.0296**	74	1.63 (0.83–3.19)	.15	84	0.57 (0.31–1.02)	.057	82	2.6 (0.86–7.9)	.081
	3 + 4	87	0.57 (0.3–1.07)	.0764	68	**1.98 (1.05–3.72**)	**.03**	88	0.59 (0.33–1.03)	.062	84	**0.45 (0.21–0.94**)	**.029**
MAP2K4	1	170	**0.41 (0.22–0.76**)	**.0034**	153	**0.41 (0.2–0.84**)	**.012**	170	**0.51 (0.27–0.95**)	**.032**	167	0.36 (0.1–1.21)	.085
	2	83	**0.11 (0.01–0.8**)	**.0081**	74	**0.45 (0.23–0.86**)	**.014**	84	**0.54 (0.3–0.98**)	**.039**	82	**0 (0-inf**)	**.015**
	3 + 4	87	1.51 (0.8–2.85)	.2051	68	**0.54 (0.29–1**)	**.045**	88	0.62 (0.36–1.07)	.082	84	2.03 (0.9–4.57)	.08
MAP2K5	1	170	1.42 (0.76–2.69)	.2715	153	**0.51 (0.29–0.91**)	**.02**	170	**0.48 (0.28–0.82**)	**.0062**	167	0.63 (0.25–1.57)	.31
	2	83	0.55 (0.25–1.18)	.1196	74	1.83 (0.74–4.56)	.19	84	1.71 (0.78–3.76)	.18	82	0.46 (0.14–1.51)	.19
	3 + 4	87	**0.37 (0.19–0.69**)	**.0014**	68	**0.34 (0.18–0.65**)	**.0007**	88	**0.36 (0.2–0.63**)	**.0003**	84	**0.27 (0.13–0.57**)	**.0002**
MAP2K6	1	170	0.76 (0.42–1.4)	.3847	153	0.67 (0.35–1.31)	.24	170	0.68 (0.37–1.24)	.2	167	0.5 (0.15–1.7)	.25
	2	83	**3.71 (1.64–8.39**)	**.0008**	74	0.69 (0.35–1.34)	.27	84	1.45 (0.78–2.68)	.24	82	**7.05 (2.11–23.51**)	**.0002**
	3 + 4	87	1.43 (0.79–2.56)	.2341	68	**1.94 (1–3.78**)	**.047**	88	1.65 (0.95–2.87)	.074	84	1.9 (0.78–4.67)	.15
MAP2K7	1	170	**2.05 (1.11–3.77**)	**.0186**	153	0.65 (0.36–1.17)	.15	170	1.32 (0.8–2.17)	.27	167	0.47 (0.2–1.15)	.091
	2	83	0.52 (0.23–1.15)	.1013	74	1.45 (0.73–2.87)	.29	84	1.41 (0.76–2.64)	.28	82	0.44 (0.15–1.36)	.14
	3 + 4	87	0.73 (0.41–1.3)	.2853	68	**0.5 (0.27–0.93**)	**.025**	88	0.61 (0.36–1.03)	.062	84	0.51 (0.25-1.03)	.054

Bold values indicate *P* < .05.

CI = confidence interval, HR = hazard ratio, OS = overall survival, PFS = progression free survival, RFS = relapse free survival.

As shown in Table 5, the increase of MAP2K1 was related to the OS and PFS of AJCC_T3, and the decrease was related to the PFS of AJCC_T2. The increase of MAP2K2 was related to the OS and DSS of AJCC_T2, and the RFS of AJCC_T3, and its decrease was related to the OS of AJCC_T1. The increase of MAP2K3 was related to the OS, RFS, PFS, and DSS of AJCC_T1, and the OS and DSS of AJCC_T3; the decrease was related to the OS of AJCC_T2 and the RFS of AJCC_T3. The elevation of MAP2K4 was related to the RFS and PFS of AJCC_T1, and the OS, RFS, PFS, and DSS of AJCC_T2. The increase of MAP2K5 was related to the RFS and PFS of AJCC_T1, and the OS, RFS, PFS, and DSS of AJCC_T3; the decrease of MAP2K6 was related to the OS and DSS of AJCC_T2, and the RFS of AJCC_T3; the increase of MAP2K7 was related to the OS of AJCC_T2, the OS of AJCC_T3 and the DSS, PFS, PFS of AJCC_T3 related, its reduction was related to the OS of AJCC_T1.

**Table 5:** Analysis of the prognostic value of MAP2Ks in LIHC patients with different AJCC_T.

MAP2Ks	AJCC_T	OS			RFS			PFS			DSS		
		CASES	HR (95% CI)	*P*	CASES	HR (95% CI)	*P*	CASES	HR (95% CI)	*P*	CASES	HR (95% CI)	*P*
MAP2K1	1	180	0.56 (0.31–1.02)	.0559	160	0.65 (0.34–1.23)	.18	180	0.63 (0.35–1.12)	.11	177	0.58 (0.25–1.34)	.2
	2	90	0.6 (0.28–1.29)	.1887	79	1.45 (0.77–2.74)	.25	92	**1.9 (1.05–3.46**)	**.032**	89	0.62 (0.23–1.69)	.35
	3	78	**0.53 (0.29–0.98**)	**.0383**	65	0.56 (0.29–1.1)	.088	78	**0.56 (0.31–1**)	**.046**	75	0.51 (0.23–1.12)	.086
	4	13	NA	NA	6	NA	NA	13	NA	NA	13	NA	NA
MAP2K2	1	180	**2.03 (1.11–3.72**)	**.0192**	160	1.42 (0.81–2.5)	.22	180	0.78 (0.43–1.4)	.4	177	1.89 (0.83–4.3)	.12
	2	90	**0.44 (0.2–0.95**)	**.0318**	79	1.71 (0.87–3.4)	.12	92	1.37 (0.74–2.54)	.32	89	**0.19 (0.05–0.67**)	**.0039**
	3	78	1.57 (0.83–2.95)	.1617	65	**0.51 (0.27–0.98**)	**.038**	78	0.62 (0.34–1.13)	.12	75	1.8 (0.84–3.87)	.13
	4	13	NA	NA	6	NA	NA	13	NA	NA	13	NA	NA
MAP2K3	1	180	**0.48 (0.27–0.86**)	**.0116**	160	**0.41 (0.24–0.7**)	**.00072**	180	**0.41 (0.25–0.68**)	**.00036**	177	**0.34 (0.15–0.78**)	**.0074**
	2	90	**2.19 (1.05–4.54**)	**.0317**	79	1.56 (0.82–2.98)	.17	92	0.58 (0.34–1.01)	.052	89	2.34 (0.89–6.14)	.077
	3	78	**0.46 (0.24–0.9**)	**.0199**	65	**2.2 (1.16–4.14**)	**.013**	78	0.56 (0.3–1.04)	.064	75	**0.36 (0.16–0.79**)	**.0079**
	4	13	NA	NA	6	NA	NA	13	NA	NA	13	NA	NA
MAP2K4	1	180	0.52 (0.28–0.94)	.0286	160	**0.41 (0.2–0.81**)	**.008**	180	**0.52 (0.29–0.93**)	**.026**	177	0.48 (0.18–1.3)	.14
	2	90	**0.19 (0.05–0.8**)	**.0117**	79	**0.46 (0.24–0.85**)	**.011**	92	**0.55 (0.32–0.95**)	**.029**	89	**0.14 (0.02–1.03**)	**.024**
	3	78	1.48 (0.76–2.89)	.2487	65	0.56 (0.3–1.05)	.069	78	0.61 (0.34–1.08)	.085	75	2.13 (0.89–5.06)	.081
	4	13	NA	NA	6	NA	NA	13	NA	NA	13	NA	NA
MAP2K5	1	180	0.75 (0.41–1.38)	.357	160	**0.46 (0.26–0.82**)	**.007**	180	**0.44 (0.26–0.76**)	**.0021**	177	0.48 (0.2–1.16)	.097
	2	90	0.61 (0.3–1.26)	.1756	79	1.82 (0.88–3.76)	.1	92	1.8 (0.95–3.41)	.067	89	0.56 (0.21–1.52)	.25
	3	78	**0.31 (0.16–0.6**)	**.00028**	65	**0.32 (0.16–0.63**)	**.00059**	78	**0.33 (0.18–0.6**)	**.00015**	75	**0.23 (0.1–0.51**)	**7.1e‐05**
	4	13	NA	NA	6	NA	NA	13	NA	NA	13	NA	NA
MAP2K6	1	180	0.75 (0.42–1.34)	.3254	160	0.65 (0.34–1.26)	.2	180	0.65 (0.36–1.17)	.15	177	0.41 (0.12–1.38)	.14
	2	90	**2.97 (1.39–6.33**)	**.0031**	79	0.74 (0.4–1.39)	.35	92	1.72(0.96–3.08)	.067	89	**4.79(1.75–13.13**)	**.00083**
	3	78	1.68 (0.91–3.1)	.0941	65	**2.04 (1.03–4.02**)	**.036**	78	1.42 (0.8–2.53)	.23	75	1.63 (0.78–3.41)	.19
	4	13	NA	NA	6	NA	NA	13	NA	NA	13	NA	NA
MAP2K7	1	180	**1.99(1.11–3.57**)	**.0183**	160	1.48 (0.87–2.51)	.14	180	1.36 (0.84–2.21)	.2	177	0.48 (0.22–1.08)	.071
	2	90	**0.48 (0.23–1.01**)	**.0049**	79	1.41 (0.73–2.71)	.3	92	1.42 (0.8–2.52)	.23	89	0.4 (0.15–1.07)	.058
	3	78	0.72 (0.39–1.32)	.2841	65	**0.42 (0.2–0.88**)	**.018**	78	**0.52 (0.29–0.91**)	**.021**	75	**0.46 (0.21–0.97**)	**.036**
	4	13	NA	NA	6	NA	NA	13	NA	NA	13	NA	NA

Bold values indicate *P* < .05.

CI = confidence interval, HR = hazard ratio, OS = overall survival, PFS = progression free survival, RFS = relapse free survival.

As shown in Table 6, the reduction of MAP2K1 was related to the PFS of microvascular invasion. MAP2K2 was not associated with the prognosis of vascular invasion in LIHC patients. The increase of MAP2K3 was related to the RFS and PFS of vascular invasion none, and the PFS of microvascular invasion, and its decrease was related to the DSS of microvascular invasion. The increase in MAP2K4/5 was related to the RFS and PFS of vascular invasion none. The increase of MAP2K5 was related to the OS of microvascular invasion. The increase of MAP2K6 was related to the PFS of vascular invasion none, and its decrease was related to the microvascular invasion OS, PFS, and DSS. The increase of MAP2K7 was related to the OS and DSS of microvascular invasion in LIHC patients.

**Table 6:** Prognostic value analysis of MAP2Ks in LIHC patients with different Vascular invasion.

MAP2Ks	Vascular invasion	OS			RFS			PFS			DSS		
		CASES	HR (95% CI)	*P*	CASES	HR (95% CI)	*P*	CASES	HR (95% CI)	*P*	CASES	HR (95% CI)	*P*
MAP2K1	None	203	0.69 (0.41–1.17)	.1712	175	1.33(0.77–2.28)	.3	204	0.77(0.47–1.25)	.29	200	1.81(0.84–3.91)	.13
	Micro	90	1.39 (0.62–3.16)	.424	81	1.52 (0.76–3.05)	.23	91	**2.11(1.18–3.79**)	**.01**	88	1.72(0.52–5.63)	.37
	Macro	16	NA	NA	14	NA	NA	16	NA	NA	14	NA	NA
MAP2K2	None	203	1.47 (0.88–2.45)	.1409	175	0.66 (0.36–1.21)	.17	204	0.72 (0.42–1.23)	.23	200	1.56 (0.73–3.37)	.25
	Micro	90	0.61 (0.24–1.52)	.2814	81	1.32 (0.7–2.49)	.38	91	0.77(0.43–1.36)	.36	88	0.27 (0.06–1.2)	.065
	Macro	16	NA	NA	14	NA	NA	16	NA	NA	14	NA	NA
MAP2K3	None	203	0.65 (0.38–1.13)	.1255	175	**0.44 (0.26–0.72**)	**.00098**	204	**0.44 (0.27–0.7**)	**.00042**	200	0.52 (0.25–1.09)	.076
	Micro	90	1.93(0.9–4.29)	.0835	81	0.55 (0.28–1.06)	.069	91	**0.47 (0.26–0.84**)	**.0097**	88	**3.19(1.07–9.51**)	**.028**
	Macro	16	NA	NA	14	NA	NA	16	NA	NA	14	NA	NA
MAP2K4	None	203	0.64 (0.37–1.11)	.1116	175	**0.52 (0.32–0.85**)	**.0073**	204	**0.54 (0.34–0.84**)	**.0061**	200	1.46(0.69–3.09)	.32
	Micro	90	0.48 (0.22–1.06)	.0644	81	1.34 (0.7–2.59)	.38	91	1.42 (0.78–2.56)	.25	88	2.88(0.63–13.15)	.15
	Macro	16	NA	NA	14	NA	NA	16	NA	NA	14	NA	NA
MAP2K5	None	203	1.34 (0.79–2.29)	.2752	175	**0.54 (0.32–0.91**)	**.018**	204	**0.55 (0.34–0.89**)	**.013**	200	0.68 (0.33–1.41)	.29
	Micro	90	**0.41 (0.19–0.88**)	**.0177**	81	1.56 (0.74–3.32)	.24	91	1.49(0.83–2.69)	.18	88	0.57 (0.19–1.76)	.33
	Macro	16	NA	NA	14	NA	NA	16	NA	NA	14	NA	NA
MAP2K6	None	203	0.71 (0.41–1.24)	.2266	175	0.61 (0.35–1.05)	.073	204	**0.58 (0.35–0.98**)	**.038**	200	0.58 (0.27–1.27)	.17
	Micro	90	**2.55(1.17–5.52**)	**.0144**	81	2.37(0.98–5.74)	.05	91	**2.5 (1.15–5.43**)	**.017**	88	**3.06 (1.02–9.21**)	**.037**
	Macro	16	NA	NA	14	NA	NA	16	NA	NA	14	NA	NA
MAP2K7	None	203	1.47 (0.86–2.5)	.1543	175	0.77(0.47–1.24)	.28	204	0.84 (0.54–1.31)	.44	200	0.57 (0.27–1.19)	.13
	Micro	90	**0.37 (0.17–0.81**)	**.0092**	81	0.66 (0.32–1.35)	.25	91	0.65 (0.36–1.16)	.14	88	**0.25(0.08–0.76**)	**.008**
	Macro	16	NA	NA	14	NA	NA	16	NA	NA	14	NA	NA

Bold values indicate *P* < .05.

CI = confidence interval, HR = hazard ratio, OS = overall survival, PFS = progression free survival, RFS = relapse free survival.

## 4. Discussion

Liver hepatocellular carcinoma is a common malignant tumor, ranking 4th in the global cause of tumor death.^[31]^ Molecular targeted therapy, as a new treatment method for patients with advanced LIHC, has become one of the hopes of breaking through the current shackles, and it is of great significance for the diagnosis, treatment, and prognosis of LIHC to find new targets.

MAP2K1 is a gene expressed in the RAS mitogen-activated protein kinase pathway. It stimulates protein kinase activity by transmitting intracellular and extracellular signals and participates in cell proliferation, differentiation, and transcriptional regulation.^[32]^ In exocrine sweat adenocarcinoma, the overexpression of MAP2K1 gene translates into strong protein expression and corresponding pathway activation in tumor tissues.^[33]^ In addition, for patients with advanced colorectal cancer, MAP2K1 mutations often indicate adverse effects of anti-EGFR therapy and MAPK pathway targeted therapy.^[34]^ Related studies believe that reducing the expression of MAP2K1 can induce cell apoptosis and inhibit HCC migration, invasion, and cell proliferation.^[35]^ Our research showed that MAP2K1 was high expression in LIHC tissues, but the protein expression was opposite. Subgroup analysis showed that it was high expression in lymphatic metastasis N0 between male and female, and low in all tumor stages and all grades. Interestingly, high expression of MAP2K1 indicates better OS. The increase of MAP2K1 was related to the OS of male patients, the RFS and PFS of Grade 1, the OS of Grade 1 to 2, the OS, RFS, PFS, and DSS of Stage 3 + 4, the OS and PFS of AJCC_T3; the decrease of MAP2K1 was related to the PFS of AJCC_T2 and Microvascular invasion-related. Our study shows that the abnormal expression of MAP2K1 may predict the occurrence of liver cancer, especially in male patients. At the same time, appropriately elevated MAP2K1 expression levels, as a therapeutic marker, may prolong OS in male patients.

MAP2K2 plays a key role in the signal transduction of mitogen growth factor.^[36]^ MAP2K2 has gain-of-function mutations in melanomas, leading to constitutive ERK phosphorylation and higher resistance to MEK inhibitors. In addition, recurrent somatic MAP2K2 mutations have been found in larger melanomas.^[37]^ This study showed that MAP2K2 was high expression in LIHC tissues, but the protein expression is opposite. Subgroup analysis shows that it is high expression between male and female, in all tumor stages, and in all grades. The prognostic value of MAP2K2 and overall LIHC patients and its correlation with the prognosis of male and female patients and Vascular invasion were not detected. However, the increase in MAP2K2 was related to the RFS and PFS of Grade3 and Stage3 + 4, the DSS of Stage2, the OS and DSS of AJCC_T2, and the RFS of AJCC_T3. The reduction of MAP2K2 was related to the OS and DSS of Grade3, the OS of Stage1/3 and AJCC_T1, and the RFS of Stage2. Our study shows that high expression of MAP2K2 can predict the occurrence of liver cancer and is positively correlated with tumor progression, but it may not be suitable as a prognostic marker.

MAP2K3 depletion can reduce cancer cell proliferation and viability.^[38]^ Xu G study proved that MAP2K3 was significantly inhibited in human HCC tumor tissues.^[36]^ By up-regulating its mRNA and protein level expression, it can promote hepatocellular carcinoma HepG2 cell proliferation.^[14]^ This study showed that MAP2K3 was under-expressed in LIHC tissues, and the protein expression was the opposite. Subgroup analysis showed low expression in all tumor stages. High expression of MAP2K3 predicts better OS, RFS, PFS, and DSS. Elevated MAP2K3 was related to the OS, RFS, PFS, and DSS of male patients, Stage1 and AJCC_T1, the PFS of female LIHC patients, Grade2 and microvascular invasion, the OS of Grade1, the RFS and PFS of Grade3 and vascular invasion none, the OS and DSS of AJCC_T3, and the DSS of Stage3 + 4. The reduction was related to the OS of Stage2 and AJCC_T2, the RFS of Stage3 + 4 and AJCC_T3, and the DSS of microvascular invasion. Our study shows that low expression of MAP2K3 can predict the occurrence of early liver cancer, and for patients with diagnosed liver cancer, high expression of MAP2K3 predicts a better prognosis. In the treatment of patients with this type of liver cancer, increasing MAP2K3 may be a more correct treatment.

MAP2K4 has a double role of inhibition and promotion in the process of tumorigenesis.^[39]^ Down-regulation of MAP2K4 can inactivate the JNK signaling pathway, thereby playing the role of tumor suppressing gallbladder cancer.^[40]^ This study showed that MAP2K4 was low expressed in LIHC tissues. The high expression of MAP2K4 indicates better OS, RFS, and PFS in overall patients. The increase of MAP2K4 was related to OS, RFS, PFS, and DSS of male patients, Stage2 and AJCC_T2. The increase in MAP2K4 was related to the OS and PFS of Grade1, the RFS and PFS of Grade2, AJCC_T1 and Vascular invasion none, the OS and RFS of Grade3, the OS, RFS and PFS of Stage1, and the RFS of Stage3 + 4. Similarly, similar to MAP2K3, low expression of MAP2K4 can predict the occurrence of liver cancer, and high expression of MAP2K4 predicts better prognosis of patients.

MAP2K5 is a new susceptibility gene for familial non-medullary thyroid cancer. Its variants A321T or M367 T can activate the MAP2K5-ERK5 pathway, thereby inducing malignant transformation of thyroid epithelial cells.^[41]^ MAP2K5 was high expression in LIHC tissues, and the protein expression was opposite. Subgroup analysis showed that it was high expression between male and female, in all tumor stages and grades. High expression of MAP2K5 indicates better OS, RFS, PFS, and DSS. Elevated MAP2K5 was related to OS, PFS, and DSS of male patients, RFS and PFS of Grade2, Stage1 and AJCC_T1, OS of Grade3 and microvascular invasion, OS, RFS, PFS, and DSS of Stage3 + 4 and AJCC_T3 in male patients.Our study shows that high expression of MAP2K5 can predict the occurrence and severity grade of liver cancer, but high expression also reflects better prognosis of patients. For this contradiction, we believe that it may be related to sample size and data bias, and cannot represent the final result, but it also shows the double-edged sword side of MAP2K5, at least it is meaningful as a predictive marker.

MAP2K6 is related to the lymph node status of bladder cancer.^[42]^ In addition, targeted down-regulation of MAP2K6 can inhibit the proliferation of esophageal adenocarcinoma cells.^[43]^ MAP2K6 was high expression in LIHC tissues, and the protein expression was consistent. Low expression of MAP2K6 is associated with better overall OS in patients with LIHC. The increase in MAP2K6 was related to the RFS and PFS of Grade1, and the PFS of Vascular invasion none. MAP2K6 reduction was related to OS, RFS and DSS of female patients, OS and DSS of Grade2, RFS and PFS of Grade3, OS and DSS of Stage2 and AJCC_T2, RFS of Stage3 + 4 and AJCC_T3, microvascular invasion OS, PFS, and DSS. Our study shows that MAP2K6 has little significance as a predictive marker, but is of great significance as a prognostic marker and therapeutic target, that is, low expression of MAP2K6 reflects a better prognosis, and lower expression of MAP2K6 can significantly prolong the survival of patients.

MAP2K7 is a therapeutic target for triple-negative breast cancer. It is down-regulating can inhibit the migration and invasion of triple-negative breast cancer Hs578T cells and hinder its epithelial–mesenchymal transition.^[44]^ MAP2K7 was high expression in LIHC tissues. Subgroup analysis showed that it was high expression between male and female and in all grades. The increase in MAP2K7 was related to OS and RFS of female patients, PFS and DSS of Grade 2, DSS of Grade 3, RFS of Stage 3 + 4, OS of AJCC_T2, RFS of AJCC_T3, PFS and DSS, and OS and DSS of microvascular invasion. Its reduction was related to the OS of Stage1 and AJCC_T1. Our study showed that MAP2K7, as a predictive marker, can well predict the occurrence of liver cancer, and as a prognostic marker, it can reflect the OS and RFS of female patients.

As a member of the MAPK pathway, the MAP2Ks family is related to the occurrence of a variety of diseases and has been studied as a potential therapeutic target for a variety of tumors. This study shows that it mainly involves functions such as protein serine/threonine kinase activity, MAP kinase kinase activity, and signaling pathways such as MAPK signaling pathway and GnRH signaling pathway. TIMER database shows that MAP2K3 to 7 were significantly related to tumor purity. Except for MAP2K3, all MAP2Ks were related to B cells. The mRNA expression of all MAP2Ks family members were significantly related to the infiltration of CD8+ T, CD4+ T cells, macrophages, neutrophils and dendritic cells.

In this study, we found that MAP2K1/2/3 was highly expressed in LIHC tissues, and the protein expression was reversed. For this contradictory finding, on the one hand, we believe that these 3 markers may have dual effects of inhibition and promotion in the tumorigenesis. In addition, it cannot be ruled out that the insufficient amount of data leads to the error of the final data. This makes us understand the shortcomings of this study. Using network database as statistical means, bioinformatics will inevitably lead to deviation of experimental results due to data sample size, authenticity of original data, bias of data entry and timeliness of data, so that the results need further experimental verification to obtain more accurate experimental results. In short, although there are some shortcomings, the results of this experiment still lay the foundation and research direction for our future research.

Next, we will verify both in vivo and in vitro experiments in order to achieve more accurate experimental results. Firstly, we will collect clinical samples to verify the mRNA and protein expression of MAP2Ks family in LIHC tissues and non-tumor liver tissues by q-PCR, Western Blot and immunohistochemistry. Then, the effect of MAP2Ks family on the proliferation and migration of hepatocellular carcinoma cells was further verified by cell experiments. Normal human liver cells HL77O2 and 4 strains of hepatocellular carcinoma cells (HCCLM3, SMMC-7721, MHCC-97H, Huh7) were selected. After culture, the experiments were further verified by real-time fluorescence quantitative PCR, Western blot, cell EdU proliferation detection, wound healing assay, transwell assay, plate cloning assay, and subcutaneous tumor formation assay in nude mice.

## 5. Conclusion

In summary, our results proved that MAP2Ks members had different patterns of expression in LIHC, and were related to gender, individual tumor stage, grade, and lymphatic metastasis status. In addition, the high expression of MAP2K1/3/4/5 and the low expression of MAP2K6 were related to the OS of LIHC patients, the high expression of MAP2K3/4/5 was related to RFS and PFS, and the high expression of MAP2K3/5/7 was related to DSS, and was related to gender, factors such as grade, stage, AJCC_T, and vascular invasion have a certain connection. In conclusion, members of the MAP2Ks family may be effective markers for the diagnostic and prognostic value of LIHC.

## Acknowledgments

The authors are grateful to the various departments of Yunnan University of Chinese Medicine for their support.

## Author contributions

**Conceptualization:** Shen Dong, Wang Huaning, Zhu Rong.

**Data curation:** Shen Dong, Shen Jing, Zhu Rong.

**Formal analysis:** Shen Dong, Shen Jing, Zhu Rong.

**Funding acquisition:** Jiao Qinshun, Wang Huaning.

**Investigation:** Shen Dong, Jiao Qinshun.

**Methodology:** Shen Dong, Shen Jing, Wang Huaning.

**Resources:** Zhu Rong.

**Software:** Shen Dong, Jiao Qinshun.

**Supervision:** Wang Huaning.

**Validation:** Shen Dong, Shen Jing, Zhu Rong.

**Visualization:** Shen Dong.

**Writing – original draft:** Shen Dong.

**Writing – review & editing:** Shen Dong, Zhu Rong.
